# Environmental scan of anal cancer screening practices: worldwide survey results

**DOI:** 10.1002/cam4.250

**Published:** 2014-04-16

**Authors:** Jigisha Patel, Irving E Salit, Michael J Berry, Alexandra de Pokomandy, Mayura Nathan, Fred Fishman, Joel Palefsky, Jill Tinmouth

**Affiliations:** 1 Sunnybrook Health Sciences CentreToronto, Canada; 2 University of TorontoToronto, Canada; 3 Toronto General HospitalToronto, Canada; 4 University of CaliforniaSan Francisco, California; 5 McGill UniversityMontreal, Canada; 6 Homerton University HospitalLondon, United Kingdom

**Keywords:** Anal cancer, HIV, screening, surveillance, treatment

## Abstract

Anal squamous cell carcinoma is rare in the general population but certain populations, such as persons with HIV, are at increased risk. High-risk populations can be screened for anal cancer using strategies similar to those used for cervical cancer. However, little is known about the use of such screening practices across jurisdictions. Data were collected using an online survey. Health care professionals currently providing anal cancer screening services were invited to complete the survey via email and/or fax. Information was collected on populations screened, services and treatments offered, and personnel. Over 300 invitations were sent; 82 providers from 80 clinics around the world completed the survey. Fourteen clinics have each examined more than 1000 patients. Over a third of clinics do not restrict access to screening; in the rest, eligibility is most commonly based on HIV status and abnormal anal cytology results. Fifty-three percent of clinics require abnormal anal cytology prior to performing high-resolution anoscopy (HRA) in asymptomatic patients. Almost all clinics offer both anal cytology and HRA. Internal high-grade anal intraepithelial neoplasia (AIN) is most often treated with infrared coagulation (61%), whereas external high-grade AIN is most commonly treated with imiquimod (49%). Most procedures are performed by physicians, followed by nurse practitioners. Our study is the first description of global anal cancer screening practices. Our findings may be used to inform practice and health policy in jurisdictions considering anal cancer screening.

## Background

Anal squamous cell carcinoma is rare in the general population, but its incidence is rising 1–3. In the United States, incidence rates are ∼1.24 per 100,000 person-years in men and 1.47 per 100,000 person-years in women 4. Rates of anal cancer are particularly elevated in HIV-infected men who have sex with men (MSM) and in other immunosuppressed persons such as transplantation recipients 2,5–8. In a systematic review the pooled incidence was reported as 46 per 100,000 person-years in HIV-infected MSM in nine studies conducted in the pre and post highly active antiretroviral therapy (HAART) eras 9. Prolonged survival due to the introduction of HAART appears to increase the risk of developing anal cancer in HIV-infected men 2. The impact of the HIV epidemic has significantly increased anal cancer incidence in men 3 perhaps partially accounting for the high incidence (131 per 100,000 person-years) reported in a recent North American cohort study 8. Currently, rates of anal cancer in HIV-infected MSM are higher than cervical cancer reported anywhere in the world 10.

Anal cancer and cervical cancer are biologically very similar; both are caused by persistent infection with high-risk types of human papillomavirus (HPV) leading to cellular oncogenic transformation at squamo-columnar junctions 11,12. In HIV-infected patients and in MSM, the prevalence of any anal HPV infection is particularly high (86–98%) 11,13–17; these patients have an increased risk of anal intraepithelial neoplasia (AIN), the precursor to anal cancer 18–20. Up to 92% of anal cancers are associated with oncogenic HPV 21–23. Because the cancers are so analogous, cervical cancer screening strategies have been applied to anal cancer. Anal cytology (also referred to as the “anal Pap smear”) and high-resolution anoscopy (HRA), which is similar to colposcopy, have been used to facilitate the detection of AIN and early invasive cancer in published literature 24. Because of the widely recognized benefits of screening for cervical cancer, there is considerable enthusiasm for anal cancer screening in high-risk populations such as HIV-infected MSM, however, little is known about current implementation of such screening practices across jurisdictions.

The objective of this study was to characterize anal cancer screening practices in jurisdictions around the world with the aims of providing clinical and operational guidance for clinicians, describing the current practice and providing information from other jurisdictions for health care policy makers.

## Methods

Clinicians and scientists currently providing anal cancer screening were invited to complete an Internet-based survey. We used a variety of strategies to contact participants from around the world. Potential respondents were identified from a review of the published literature, conference abstract books, and list of physicians who had been trained in HRA provided by the American Society for Colposcopy and Cervical Pathology (ASCCP). To our knowledge, the ASCCP is the only organization that provides formal training in HRA in the world. As such, our recruitment strategy should have identified a high percentage of clinicians who are practicing in this field.

Participants were invited to complete the survey via email and/or fax in April 2011. Survey questions were deployed using a web-based survey tool (Survey Monkey™, Palo Alto, CA). Participants were asked to answer up to 40 questions about their practice and their clinic or program. Information was collected on populations screened, services and treatments offered, and personnel. Respondent data were exported from Survey Monkey and descriptive analyses (counts, percentages/proportions) were performed.

## Results

Over 300 email/fax invitations were sent; 82 providers from 80 clinics in Canada, USA, Europe, Asia, and Australia completed the survey.

### Clinic/program

**Figure 1:**
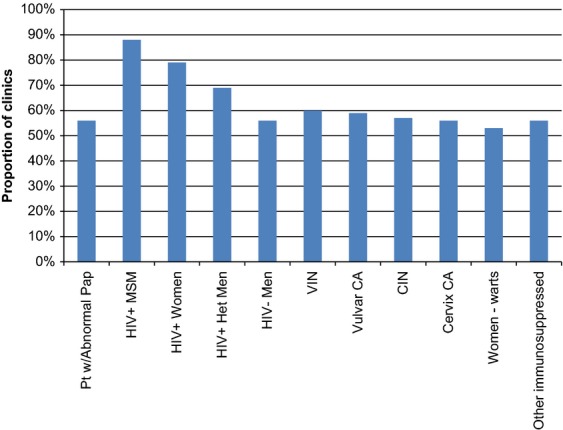
Characteristics of populations accepted by the anal neoplasia screening clinics/programs, reported as proportion of clinics seeing each population (*n* = 80)^a^. CA, cancer; CIN, cervical intraepithelial neoplasia; Het, heterosexual; MSM, men who have sex with men; Pt w/, patient with; VIN, vulvar intraepithelial neoplasia; warts, external genital warts. ^a^Respondents were allowed to select more than one option.

Most clinics are relatively new and consequently, have only provided care to a small volume of patients. While 34 (43%) clinics have examined fewer than 100 patients, 14 (18%) clinics have examined more than 1000 patients since their inception (Table1). Almost all clinics offer anal cytology (98%) and HRA (99%). Treatment of high-grade (89%) and low-grade (84%) AIN is also offered in most clinics, however, just 59% of the clinics offer HPV testing using either PCR, hybrid capture, or both. Physicians, followed by nurse practitioners, most commonly performed the procedures (Table2). Approximately 1/3 of clinics do not risk stratify (i.e., anyone is eligible for their services). Most clinics will see HIV-infected persons, regardless of sex or sexual orientation. Just over 50% of clinics offer their services to other high-risk groups (those with abnormal Pap, women with HPV, other immunosuppressed individuals) (Fig.1).

**Table 1:** Characteristics of clinics that responded to the survey (*n* = 80).

Country/region	Number of clinics responded	Number of clinics part of a larger entity (vs. stand alone clinics)	Number of years clinic has been providing services^1^				Number of patients seen in clinic since inception^1^				
			0–2	>2–5	>5–10	>10–20	0–100	101–250	251–500	501–1000	>1000
USA	62	48 (14)	25	21	10	5	30	9	7	6	9
Canada	5	5 (0)	1	1	3	–	2	–	–	3	–
Australia	3	3 (0)	–	–	2	1	–	–	1	1	1
United Kingdom	3	3 (0)	–	–	-	3	–	–	–	2	1
Italy	1	1 (0)	–	–	–	1	1	–	–	–	–
Spain	4	4 (0)	2	1	1	–	–	–	2	–	2
Thailand	1	1 (0)	–	1	–	–	–	–	–	–	1
Puerto Rico	1	0 (1)	1	–	–	–	1	–	–	–	–
Total	80	65 (15)	29	24	16	10	34	9	10	12	14

1 One USA clinic is missing information.

**Table 2:** Anal cancer screening-related services offered by respondents.

	Proportion of clinics offering the service (%)	Health professional doing the procedure by proportion of clinics (%)		
Physician		Nurse practitioner	Other^2^
Screening-related services				
Anal cytology	98	87	60	30
HPV testing using hybrid capture^1^	41	34	23	11
HPV testing using PCR^1^	36	29	11	11
Vaccine against HPV	51	N/A		
Procedures/treatment-related services				
HRA	99	84	35	4
Treatment of LG AIN	84	76	28	4
Treatment of HG AIN	89	82	23	4
Treatment of anal warts/condyloma	86	81	46	15
HRA guided surgery, including WLE	31	N/A		
Other				
STD screening/testing	76	N/A		
Follow-up of patients with a prior history of anal cancer/neoplasia	81	N/A		

Results reported by proportion of clinics/programs offering the procedure. AIN, anal intraepithelial neoplasia; HRA, high-resolution anoscopy; LG/HG, low/high grade; N/A, not asked; WLE, wide local excision.

1 Some clinics offered both hybrid capture and PCR HPV testing.

2 Other, other health care professional including nurses.

### Screening/diagnosis

Fifty-three percent of clinics will perform HRA in asymptomatic patients only if the anal cytology result is abnormal. Of these clinics, 74% use a cytology result of abnormal squamous cells of uncertain significance (ASC-US) or worse to trigger HRA, whereas 24% use low-grade squamous intraepithelial lesion (LSIL) or higher and 2% use a threshold of high-grade squamous intraepithelial lesion (HSIL). When performing HRA, 68% of respondents routinely do anal cytology concurrently while 32% of physicians routinely do HPV testing concurrently. Seventy-six percent routinely assess persons with external high-grade AIN, including Bowen's disease, for the presence of internal AIN with HRA and guided biopsies, whereas only 28% of clinics routinely perform mapping or systematic four-quadrant biopsies to assess the extent of their external disease.

### Treatment

**Figure 2:**
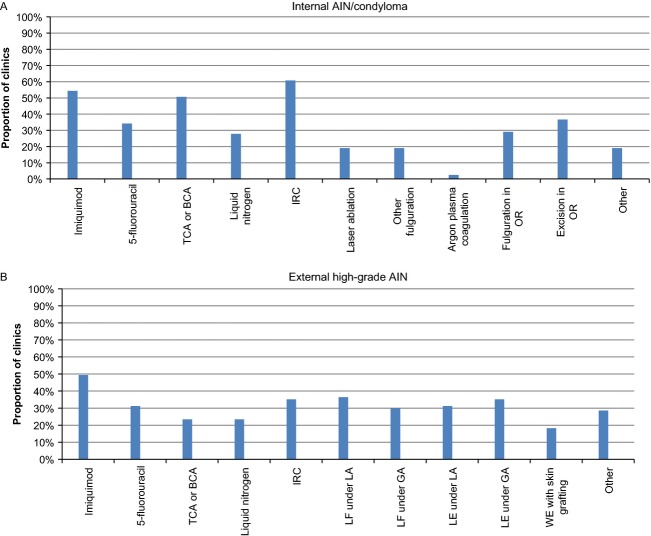
Type of treatments offered in clinics/programs for internal (A) and external (B) anal intraepithelial neoplasia (AIN), reported as the proportion of clinics offering each treatment. IRC, infrared coagulation; LA or GA, local or general anesthesia; LE, local excision; LF, local ablation/fulguration; OR, operating room; TCA or BCA, trichloroacetic acid or bichloroacetic acid; WE, wide excision.

Different therapeutic approaches were used for the treatment of internal and external AIN. The most commonly used treatment for internal disease is infrared coagulation (IRC) (61%) followed by imiquimod (54%) and bi/trichloroacetic acid (51%). External high-grade disease is most commonly treated with imiquimod (49%) followed by local fulguration strategies, including but not limited to IRC (Fig.2A and B).

### Surveillance

**Figure 3:**
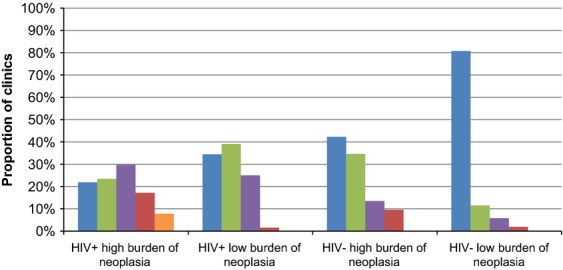
Respondents' estimations of the risk of posttreatment recurrence of high-grade dysplasia stratified by baseline risk. Estimated risk of recurrence: 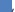 0–20%; 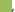 21–40%; 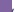 41–60%; 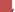 61–80%; 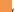 81–100%.

Respondents were asked to estimate the risk of recurrence of high-grade AIN after treatment in their clinics in various clinical scenarios. HIV-infected patients with a high burden of AIN were estimated to have the highest risk of recurrence of their high-grade disease (Fig.3). Practitioners appear to rely on severity of AIN and to a lesser extent on immune status when planning the interval to the next HRA surveillance. In patients with a diagnosis of anal cancer in the last 2–5 years, most respondents recommended repeat surveillance within 1 year for immunocompetent patients and within 6 months for immunosuppressed patients. In all other scenarios, recommended time of repeat HRA surveillance appears to be independent of patients' HIV status (Table3).

**Table 3:** Interval recommended by 50% or more respondents for repeat HRA surveillance in (A) immunocompetent and (B) immunosuppressed patients stratified by baseline findings.

Scenario	(A) Immunocompetent patients	(B) Immunosuppressed patients
Negative anal Pap smear, negative HPV, negative high-resolution anoscopy	Within 2 years	
Low-grade dysplasia on anal pap smear, negative high-resolution anoscopy	Within 1 year	
High-grade dysplasia on anal pap smear, negative high-resolution anoscopy	Within 6 months	
Histologic low-grade dysplasia	Within 1 year	
Histologic high-grade dysplasia	Within 6 months	
Diagnosis of anal cancer in last 2 years	Within 6 months	
Diagnosis of anal cancer >2 years but <5 years ago	Within 1 year	Within 6 months
Diagnosis of anal cancer >5 years ago	Within 1 year	

HRA, high-resolution anoscopy; HPV, human papillomavirus.

### Personnel and training

The majority of clinics have dedicated supporting services such as colorectal surgeons (69%), cytologists (66%), and histopathologists (66%). Most providers learned HRA by attending a formal course (82%) and/or by apprenticing with an experienced colleague (79%). Forty-four percent of providers train other personnel to do HRA.

### Payment/funding

**Figure 4:**
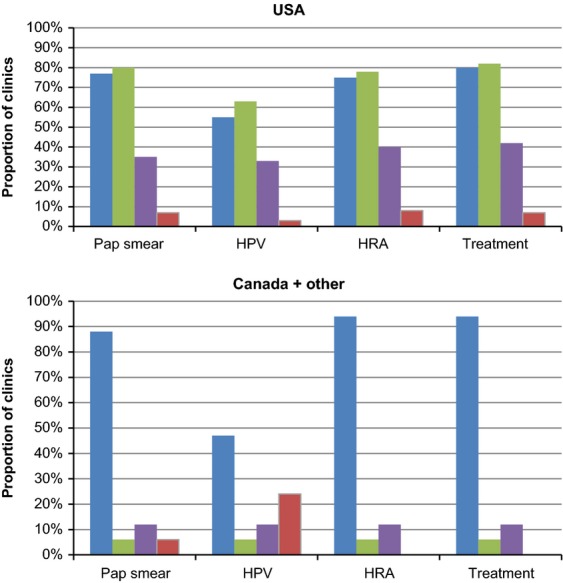
Methods of payment for various anal cancer screening tests, reported by number of clinics^a^. 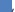, publicly funded health coverage; 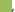, private insurance; 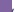, patients; 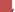, others. ^a^More than one funding source may be reported.

**Figure 5:**
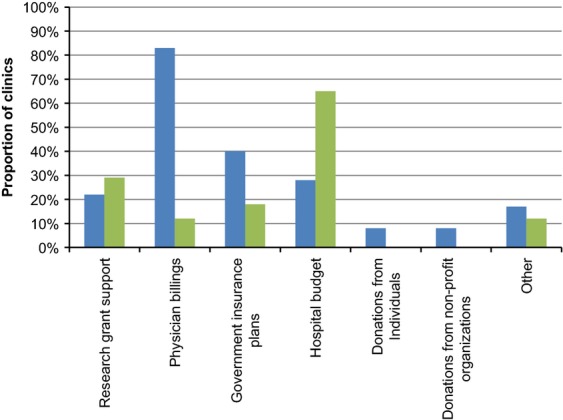
Funding source for clinic/program, reported by proportion of clinics. ^a^More than one funding source may be reported. 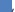, USA; 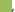, others.

Private insurance, closely followed by public funds, are the two most common methods of payment for anal cancer screening tests in the United States, whereas in Canada and other countries public funds are almost exclusively used for payment (Fig.4). In the United States, most clinics (83%) are funded by physician billings (i.e., insurance companies or health maintenance organizations [HMOs]), while the majority of clinics in other parts of the world receive funds from hospital budgets (65%) (Fig.5).

## Discussion

Our study is the first to describe current anal cancer screening practices globally. Just over half of the clinics that responded require that patients have an abnormal anal cytology result in order to proceed to HRA; the rest are performing HRA directly. Most clinics restrict their services to selected high-risk populations. Treatment of both high-grade and low-grade AIN is also offered in most clinics, however, less than two third of clinics offer HPV testing. Our respondents used ablative therapy most commonly for internal AIN and patient applied topical treatment for external AIN. However, there was considerable variation in the types of treatment offered for both internal and external AIN, suggesting either a lack of consensus about the best treatment strategy among our respondents or differences in respondent expertise and access to the necessary equipment. Severity of AIN appears to be the principal factor in determining the interval respondents recommend between surveillance visits.

We found that almost half of clinics do not require an abnormal cytology result in order to proceed to HRA. To date, no long-term controlled trials have been performed to evaluate anal cancer screening; hence, practitioners often rely on the approaches used for cervical cancer screening 25. In contrast to our findings, many guidelines 26,27 for cervical cancer screening indicate that a screening test, usually a cervical Pap smear, should be done prior to colposcopy. Similarly, experts often recommend that anal cytology should be the first step in anal cancer screening; those patients with abnormal cytology results would then be further evaluated by HRA 7,10,24. The data from several studies 11,28–32 suggest that the sensitivity of anal cytology for the detection of biopsy-proven high-grade AIN in HIV-infected men is comparable to the sensitivity of cervical cytology for the detection of cervical high-grade disease. Despite these data, our respondents commonly reported direct use of HRA, rather than risk stratifying with cytology. This could partly be explained by a preference to do cytology at the time of HRA as a form of quality control for HRA findings, rather than as part of determining eligibility for HRA. While direct HRA is a resource intensive practice, interestingly, it was recently found to be the most cost-effective strategy for the detection of high-grade AIN in HIV-infected MSM 33. The optimal approach to screening for anal cancer is not known; well-controlled prospective studies would help to guide clinical practice.

We found relatively low rates of HPV testing in our sample. MSM and HIV-infected individuals have high rates of anal cancer 2 and not surprisingly, the prevalence of HPV infection is also especially high in these groups (86–98%) 11,13–17. As a result, anal HPV testing is not likely to be useful for risk stratification in these high-risk populations, as it is for cervical cancer screening in HIV-negative women where HPV infection is less common 34.

High-grade AIN is difficult to treat—recurrence is common, especially in immunocompromised populations where there is a higher likelihood of HPV persistence. According to our survey, IRC is most frequently used in clinical practice, particularly for the treatment of internal AIN. Goldstone et al. 35,36 have performed retrospective reviews of the use of IRC for treating anal high-grade squamous AIN. Although recurrence was high after the first IRC, repeated treatment led to resolution of high-grade AIN, with a 72% probability of cure in retreated lesions in HIV-infected MSM. They report that IRC is a safe and effective procedure for treatment in both HIV-infected MSM and HIV-negative patients; this was further confirmed in a prospective pilot study by Stier et al. 37. The second most common treatment used by our respondents is imiquimod. Several uncontrolled studies have found that imiquimod is a safe, effective, and well-tolerated treatment option for AIN in HIV-infected patients, with response rates ranging from 46% to 74% 38–40. In contrast, a more recent study 41 showed electrocautery to be more effective and tolerable than topical imiquimod or 5-fluorouracil as a treatment for AIN in HIV-infected MSM, 57% of whom had high-grade disease. Despite these encouraging data, little is known about the long-term response rates. Larger studies with long-term follow-up are necessary to determine the most effective and most durable treatment options.

External high-grade AIN, including perianal Bowen's disease, poses its own challenges. It is prone to local recurrence and treatment remains controversial 42,43. According to the literature, surgical excision appears to be the therapy of choice for Bowen's disease 44–48. In a survey of American colorectal surgeons, Cleary et al. 46 found that most surgeons advocate wide local excision for macroscopic small (96%) and large (87%) lesions, while favoring a more conservative option (i.e., observation alone) (74%) for patients with microscopic disease. In this study, however, respondents to our survey indicated that they used imiquimod more commonly than wide excision to treat Bowen's disease. The reasons for this discrepancy between current clinical practice and the literature are unclear and may include perceived severity of the disease, the specialty of the treating physician or the preferences of their surgical colleagues. The literature supporting surgical remedies is relatively old; and, as also reported for high-grade vulvar intraepithelial neoplasia (VIN) 49,50, there are recent case reports that support the use of imiquimod for Bowen's disease 42,51. However, there have been no comparative effectiveness studies of different treatments for Bowen's disease 42,46.

The optimal surveillance protocol for persons with AIN is unknown. When recommending an interval to repeat surveillance, the majority of respondents in our study relied on the severity of the AIN and to a lesser extent, on immune status. Recommendations reported by our respondents appear to be consistent with an algorithm proposed by Abbasakoor and Boulos 52, which recommends a clinical review every 12 months in HIV-negative patients with low-grade AIN and every 4–6 months in HIV-infected or patients with high-grade AIN. Palefsky 53 recommends a slightly more aggressive schedule—clinical and cytological screening every 12 months in HIV-infected patients, every 6 months for those with low-grade AIN and every 3 months in patients with high-grade AIN. Cost-effectiveness studies by Goldie et al. 54,55 recommend screening HIV-infected MSM annually and biennial screening of HIV-negative MSM. These recommendations, however, are based on limited evidence about the long-term durability of treatment and the natural history of high-grade AIN, especially in HIV-infected persons on antiretrovirals. Therefore, studies to determine the most appropriate surveillance protocol are required.

Despite the relatively large number of clinicians who responded, our sample may still not be entirely representative. Efforts were made to be as inclusive as possible when identifying potential respondents by capitalizing on all existing resources available to us, including a literature review to identify persons who had published in the field. As a result, our response rate appears low, but this likely reflects the fact that our recruitment strategy included a high proportion of persons who do not perform HRA. To our knowledge, the ASCCP is the only organization that provides formal training in HRA in the world. Abbreviated introductory courses in HRA have also been sponsored by the American Society of Colon and Rectal Surgeons (ASCRS) and the American College of Surgeons (ACS) via the ASCCP, but these organizations were not surveyed. Even so, our recruitment strategy should have identified most clinicians practicing in this field. However, we may have missed some clinicians, such as those who are trained by colleagues, for example, and not through a formal course. Nonetheless, we have responses from a large number of anal cancer screening clinics from around the world thus providing an arguably global perspective on the practice of anal cancer screening.

We have demonstrated considerable variation in anal cancer screening practice; there appears to be no universal consensus on optimal strategies for anal cancer screening, treatment, and follow-up. In large part, this is due to the lack of prospective controlled studies or well-designed observational studies. The incidence of anal cancer worldwide continues to increase likely in part because progression to invasive anal cancer from AIN is slow and HIV-infected individuals are now living longer. Hence, there is a need for rigorous studies to identify the most effective screening and treatment strategies in populations at high risk for anal cancer such as HIV-infected MSM. In the interim, the variation in practice that we have demonstrated is concerning and suggests the need for a formal consensus process whereby screening for, diagnosis and treatment of AIN is standardized using best available evidence and expert opinion.

## Conflict of Interest

None declared.

## References

[b1] Tinmouth J, Paszat L, Rabeneck L (2006). Anal cancer in Ontario, 1971-2002: the relationship to socioeconomic status, sex and the HIV epidemic. Am. J. Gastroenterol.

[b2] D'Souza G, Wiley DJ, Li X, Chmiel JS, Margolick JB, Cranston RD (2008). Incidence and epidemiology of anal cancer in the multicenter AIDS cohort study. J. Acquir. Immune Defic. Syndr.

[b3] Shiels MS, Pfeiffer RM, Chaturvedi AK, Kreimer AR, Engels EA (2012). Impact of the HIV epidemic on the incidence rates of anal cancer in the United States. J. Natl. Cancer Inst.

[b4] Cook MB, Dawsey SM, Freedman ND, Inskip PD, Wichner SM, Quraishi SM (2009). Sex disparities in cancer incidence by period and age. Cancer Epidemiol. Biomarkers Prev.

[b5] Diamond C, Taylor TH, Aboumrad T, Bringman D, Anton-Culver H (2005). Increased incidence of squamous cell anal cancer among men with AIDS in the era of highly active antiretroviral therapy. Sex. Transm. Dis.

[b6] Patel P, Hanson DL, Sullivan PS, Novak RM, Moorman AC, Tong TC (2008). Incidence of types of cancer among HIV-infected persons compared with the general population in the United States, 1992-2003. Ann. Intern. Med.

[b7] Chiao EY, Giordano TP, Palefsky JM, Tyring S, El Serag H (2006). Screening HIV-infected individuals for anal cancer precursor lesions: a systematic review. Clin. Infect. Dis.

[b8] Silverberg MJ, Lau B, Justice AC, Engels E, Gill MJ, Goedert JJ (2012). Risk of anal cancer in HIV-infected and HIV-uninfected individuals in North America. Clin. Infect. Dis.

[b9] Machalek DA, Poynten M, Jin F, Fairley CK, Farnsworth A, Garland SM (2012). Anal human papillomavirus infection and associated neoplastic lesions in men who have sex with men: a systematic review and meta-analysis. Lancet Oncol.

[b10] Palefsky JM (2009). Anal cancer prevention in HIV-positive men and women. Curr. Opin. Oncol.

[b11] Salit IE, Lytwyn A, Raboud J, Sano M, Chong S, Diong C (2010). The role of cytology (Pap tests) and human papillomavirus testing in anal cancer screening. AIDS.

[b12] Coutlee F, de Pokomandy A, Franco EL (2012). Epidemiology, natural history and risk factors for anal intraepithelial neoplasia. Sex. Health.

[b13] de Pokomandy A, Rouleau D, Ghattas G, Vézina S, Coté P, Macleod J (2009). Prevalence, clearance, and incidence of anal human papillomavirus infection in HIV-infected men: the HIPVIRG cohort study. J. Infect. Dis.

[b14] Kreuter A, Brockmeyer NH, Hochdorfer B, Weissenborn SJ, Stücker M, Swoboda J (2005). Clinical spectrum and virologic characteristics of anal intraepithelial neoplasia in HIV infection. J. Am. Acad. Dermatol.

[b15] Palefsky JM, Holly EA, Ralston ML, Jay N (1998). Prevalence and risk factors for human papillomavirus infection of the anal canal in human immunodeficiency virus (HIV)-positive and HIV-negative homosexual men. J. Infect. Dis.

[b16] Palefsky JM, Holly EA, Efirdc JT, Da Costa M, Jay N, Berry JM (2005). Anal intraepithelial neoplasia in the highly active antiretroviral therapy era among HIV-positive men who have sex with men. AIDS.

[b17] Critchlow CW, Hawes SE, Kuypers JM, Goldbaum GM, Holmes KK, Surawicz CM (1998). Effect of HIV infection on the natural history of anal human papillomavirus infection. AIDS.

[b18] Scholefield JH, Castle MT, Watson NFS (2005). Malignant transformation of high-grade anal intraepithelial neoplasia. Br. J. Surg.

[b19] Watson AJM, Smith BB, Whitehead MR, Sykes PH, Frizelle FA (2006). Malignant progression of anal intra-epithelial neoplasia. ANZ J. Surg.

[b20] Kreuter A, Potthoff A, Brockmeyer NH, Gambichler T, Swoboda J, Stücker M (2010). Anal carcinoma in human immunodeficiency virus-positive men: results of a prospective study from Germany. Br. J. Dermatol.

[b21] Frisch M, Fenger C, van den Brule AJC, Sørensen P, Meijer CJLM, Walboomers JMM (1999). Variants of squamous cell carcinoma of the anal canal and perianal skin and their relation to human papillomaviruses. Cancer Res.

[b22] Daling JR, Madeleine MM, Johnson LG, Schwartz SM, Shera KA, Wurscher MA (2004). Human papillomavirus, smoking, and sexual practices in the etiology of anal cancer. Cancer.

[b23] Parkin DM BF (2006). Chapter 2: the burden of HPV-related cancers. Vaccine.

[b24] Tinmouth J, Raboud J, Ali M, Malloch L, Su D, Sano M (2011). The psychological impact of being screened for anal cancer in HIV-infected men who have sex with men. Dis. Colon Rectum.

[b25] Sigel K, Dubrow R, Silverberg M, Crothers K, Braithwaite S, Justice A (2011). Cancer screening in patients infected with HIV. Curr. HIV/AIDS Rep.

[b26] USPSTF (2012). http://www.uspreventiveservicestaskforce.org/uspstf11/cervcancer/cervcancerrs.htm.

[b27] Canadian Task Force on Preventive Health Care (2013). Recommendations on screening for cervical cancer. CMAJ.

[b28] Palefsky JM, Holly EA, Hogeboom CJ, Berry JM, Jay N, Darragh TM (1997). Anal cytology as a screening tool for anal squamous intraepithelial lesions. J. Acquir. Immune. Defic. Syndr. Hum. Retrovirol.

[b29] Lee A, Young T, Hanks D, Ung R, Stansell J (2004). The evaluation of anal dysplasia with anal cytology (Pap) followed by high resolution anoscopy (HRA) and biopsy in HIV-infected men. Int. Conf AIDS.

[b30] Mathews WC, Sitapati A, Caperna J, Barber RE, Tugend A, Go U (2004). Measurement characteristics of anal cytology, histopathology, and high-resolution anoscopic visual impression in an anal dysplasia screening program. J. Acquir. Immune Defic. Syndr.

[b31] Panther LA, Wagner K, Proper J, Fugelso DK, Chatis PA, Weeden W (2004). High resolution anoscopy findings for men who have sex with men: inaccuracy of anal cytology as a predictor of histologic high-grade anal intraepithelial neoplasia and the impact of HIV serostatus. Clin. Infect. Dis.

[b32] Fox PA, Seet JE, Stebbing J, Francis N, Barton SE, Strauss S (2005). The value of anal cytology and human papillomavirus typing in the detection of anal intraepithelial neoplasia: a review of cases from an anoscopy clinic. Sex. Transm. Infect.

[b33] Lam JMC, Hoch JS, Tinmouth J, Sano M, Raboud J, Salit IE (2011). Cost-effectiveness of screening for anal precancers in HIV-positive men. AIDS.

[b34] Saslow D, Solomon D, Lawson HW, Killackey M, Kulasingam SL, Cain JM (2012). American Cancer Society, American Society for Colposcopy and Cervical Pathology, and American Society for Clinical Pathology screening guidelines for the prevention and early detection of cervical cancer. J. Low. Genit. Tract Dis.

[b35] Goldstone SE, Kawalek AZ, Huyett JW (2005). Infrared coagulator: a useful tool for treating anal squamous intraepithelial lesions. Dis. Colon Rectum.

[b36] Goldstone S, Hundert J, Huyett J (2007). Infrared coagulator ablation of high-grade anal squamous intraepithelial lesions in HIV-negative males who have sex with males. Dis. Colon Rectum.

[b37] Stier EA, Goldstone SE, Berry JM, Panther LA, Jay N, Krown SE (2008). Infrared coagulator treatment of high-grade anal dysplasia in HIV-infected individuals: an aids malignancy consortium pilot study. J. Acquir. Immune Defic. Syndr.

[b38] Wieland U, Brockmeyer NH, Weissenborn SJ, Hochdorfer B, Stucker M, Swoboda J (2006). Imiquimod treatment of anal intraepithelial neoplasia in HIV-positive men. Arch. Dermatol.

[b39] Sanclemente G, Herrera S, Tyring SK, Rady PL, Zuleta JJ, Correa LA (2007). Human papillomavirus (HPV) viral load and HPV type in the clinical outcome of HIV-positive patients treated with imiquimod for anogenital warts and anal intraepithelial neoplasia. J. Eur. Acad. Dermatol. Venereol.

[b40] Kreuter A, Potthoff A, Brockmeyer NH, Gambichler T, Stucker M, Altmeyer P (2008). Imiquimod leads to a decrease of human papillomavirus DNA and to a sustained clearance of anal intraepithelial neoplasia in HIV-infected men. J. Invest. Dermatol.

[b41] Richel O, de Vries H, van Noesel C, Dijkgraaf M, Prins J (2012).

[b42] van Egmond S, Hoedemaker C, Sinclair R (2007). Successful treatment of perianal Bowen's disease with imiquimod. Int. J. Dermatol.

[b43] Cleary RK, Schaldenbrand JD, Fowler JJ, Schuler JM, Lampman RM (1999). Perianal Bowen's disease and anal intraepithelial neoplasia: review of the literature. Dis. Colon Rectum.

[b44] Sarmiento JM, Wolff BG, Burgart LJ, Frizelle FA, Ilstrup DM (1997). Perianal Bowen's disease: associated tumors, human papillomavirus, surgery, and other controversies. Dis. Colon Rectum.

[b45] Marchesa P, Fazio VW, Oliart S, Goldblum JR, Lavery IC (1997). Perianal Bowen's disease: a clinicopathologic study of 47 patients. Dis. Colon Rectum.

[b46] Cleary RK, Schaldenbrand JD, Fowler JJ, Schuler JM, Lampman RM (2000). Treatment options for perianal Bowen's disease: survery of American Society of Colon and Rectal Surgeons Members. Am. Surg.

[b47] Cox NH, Eedy DJ, Morton CA (1999). Guidelines for management of Bowen's disease. British Association of Dermatologists. Br. J. Dermatol.

[b48] Cox NH, Eedy DJ, Morton CA (2007). Therapy Guidelines and Audit Subcommittee BAoD. Guidelines for management of Bowen's disease: 2006 update. Br. J. Dermatol.

[b49] Mahto M, Nathan M, O'Mahony C (2010). More than a decade on: review of the use of imiquimod in lower anogenital intraepithelial neoplasia. Int. J. STD AIDS.

[b50] Iavazzo C, Pitsouni E, Athanasiou S, Falagas ME (2008). Imiquimod for treatment of vulvar and vaginal intraepithelial neoplasia. Int. J. Gynecol. Obstet.

[b51] Gutzmer R, Kaspari M, Vogelbruch M, Kiehl P, Kapp A, Werfel T (2002). Successful treatment of anogenital Bowen's disease with the immunomodulator imiquimod, and monitoring of therapy by DNA image cytometry. Br. J. Dermatol.

[b52] Abbasakoor F, Boulos PB (2005). Anal intraepithelial neoplasia. Br. J. Surg.

[b53] Palefsky JM (2000). Anal squamous intraepithelial lesions in human immunodeficiency virus-positive men and women. Semin. Oncol.

[b54] Goldie S, Kuntz K, Weinstein M, Freedberg K, Welton M, Palefsky JM (1999). The clinical effectiveness and cost-effectiveness of screening for anal squamous intraepithelial lesions in homosexual and bisexual HIV-positive men. JAMA.

[b55] Goldie SJ, Kuntz KM, Weinstein MC, Freedberg KA, Palefsky JM (2000). Cost-effectiveness of screening for anal squamous intraepithelial lesions and anal cancer in human immunodeficiency virus-negative homosexual and bisexual men. Am. J. Med.

